# The value of tranexamic acid for patients with preoperative anemia in primary total knee arthroplasty

**DOI:** 10.1186/s40001-019-0387-4

**Published:** 2019-08-12

**Authors:** Minwei Zhao, Xiao Geng, Cheng Wang, Lin Zeng, Hua Tian

**Affiliations:** 1 Department of Orthopedics, Peking University Third Hospital, No 49 Huayuan Road, Haidian District, Beijing, People’s Republic of China; 2 Epidemiology Center, Peking University Third Hospital, Beijing, People’s Republic of China

**Keywords:** Preoperative anemia, Tranexamic acid, Total knee arthroplasty, Hemostasis

## Abstract

**Background:**

Preoperative anemia is relatively common in patients undergoing primary total knee arthroplasty (TKA), and is associated with higher medical costs due to blood transfusions.

**Methods:**

We aimed to discuss the efficiency of tranexamic acid (TXA) administration for blood loss control in preoperatively anemic patients undergoing primary TKA. We retrospectively reviewed the clinical data regarding a consecutive series of primary unilateral TKA patients with preoperative anemia. Patients were divided into TXA group (received TXA peri-operatively) and control group (did not receive TXA). Outcome measures included drainage volume; hemoglobin, and hematocrit levels recorded preoperatively and over the first 5 days postoperatively; amount of allogeneic blood transfusion; and prevalence of thrombosis.

**Results:**

Ninety-six patients from 996 cases were included in the study. Demographics, general health condition, and preoperative conditions were comparable between the two groups. However, significantly lower drainage volume (*P *< 0.001), hidden blood loss (*P *< 0.001), and allogeneic blood transfusion volume (*χ *= 4.00, *P *= 0.046) were noted in TXA group. The hemoglobin and hematocrit levels were significantly higher in TXA group on the first postoperative day (*P *= 0.006), but overall the drop in hemoglobin and hematocrit levels over the first 5 days postoperatively was similar between the groups (*P *= 0.763), as was the incidence of thromboembolism events (*P *= 0.794).

**Conclusion:**

TXA has a positive role for patients with preoperative anemia in primary total knee arthroplasty. In patients with mild preoperative anemia, TXA decreases hidden blood loss and the need for allogeneic blood transfusion, which mainly appears effective on the first postoperative day of TKA.

## Introduction

Total knee arthroplasty (TKA) is a successful surgical intervention for treating end-stage osteoarthritis, and encouraging reports regarding long-term clinical outcomes have been published [1–3]. Approximately 350,000 TKAs are performed annually in the USA [4]; in mainland China, the number of procedures performed each year has been increasing steadily.

Approximately 24% of patients undergoing elective total joint arthroplasty have preoperative anemia [5], and, as a result, require a significant amount of allogeneic blood transfusion (ABT) [6], which is sometimes associated with prolonged hospital stay and poor clinical outcome [7, 8].

The application of the antifibrinolytic agent tranexamic acid (TXA) in TKA may significantly decrease total blood loss and the need for transfusion without increasing the incidence of venous thromboembolism for non-anemic patients [9–11]. However, a few studies have focused on the effects of TXA in patients with preoperative anemia undergoing TKA [12]. Therefore, we designed the present study to perform a preliminary evaluation of the efficiency and safety of TXA for TKA patients with preoperative anemia.

## Materials and methods

### Patients

We retrospectively reviewed the clinical data regarding consecutive patients who underwent primary unilateral TKA in our institution between June 2013 and December 2015. The inclusion criteria were anemia during the month before surgery, defined, according to the anemia standard put forward by the World Health Organization (WHO), as preoperative hemoglobin (Pre-op Hb) levels < 13 g/dL for males and < 12 g/dL for females; physical status level of 1 or 2, as defined by the American Society of Anesthesiologists; and Charlson comorbidity score < 6. The exclusion criteria were hemorrhagic disease; previous history of surgical infection in the affected limb; history of malignant tumor; and the patients who received TXA before 2015, because of the varied methods of drug use.

### Surgical procedure and postoperative management

The patient received lumbar anesthesia, and surgery was performed with a midline skin incision and medial parapatellar approach. A pneumatic tourniquet was inflated at 300–320 mmHg, and maintained under inflated condition throughout the duration of the surgery. Total condylar prosthesis with an open design (Genesis II; Smith & Nephew, Memphis, TN, USA) was implanted in all cases. None of the patients received patella resurfacing. The whole status of equipment of the hospital, operative theater, and anesthesia medication were not different during the study period from 2013 to 2015. Drainage was performed in every patient for 24 h after surgery, and an elastic bandage was used during this period to create localized pressure. Prophylaxis for thromboembolism was performed by means of rivaroxaban (10 mg/day), and early function rehabilitation was carried out routinely in all patients. Lower extremity venous ultrasound was performed on the fifth day after surgery to identify potential deep venous thrombosis (DVT) events.

A standard protocol for administration of TXA was established in our institute at the beginning of 2015, and thereafter all patients undergoing primary unilateral TKA received TXA unless contraindicated because of the following conditions: renal insufficiency(blood creatinine levels > 133 μmol/L), hepatic insufficiency, severe respiratory disease, cardiovascular disease, coronary stent implantation during the year before TKA, coagulopathy, high risk of thrombosis (congenital/acquired thrombotic diseases), history of venous thromboembolism, or history of stroke.

The study population was divided into two groups. The TXA group was defined as containing patients who received intravenous TXA (Guangzhou Pharmaceutical Group, Guangzhou, China; batch number: H20056987; specification: 10 mL: 1.0 g), the administration of TXA was 1.0 g at 15 min before inflation of the tourniquet, and 1.0 g after tourniquet release postoperatively. The patients who did not receive TXA were included in the control group. ABT was indicated for patients with Hb < 80 g/L, hematocrit (HCT) < 20%, or other obvious anemia manifestations.

### Outcome measures

The following data were recorded: baseline characteristics including demographic data (age, sex) and general information regarding the health condition (body mass index, American Society of Anesthesiologists physical status score, Charlson score); preoperative Hb, HCT, and creatinine clearance levels; tourniquet time; postoperative Hb and HCT levels over the first 5 days after surgery; postoperative drainage volume; and volume of ABT needed for each patient.

The Gross equation was used to calculate the amount of hidden blood loss (HBL). First, the total blood loss (mL) was calculated as preoperative blood volume × (preoperative HCT level − postoperative HCT level). Preoperative blood volume was calculated by the Nadler method as k1 × H3 + k2 × W+k3, where * H* is the height (m), *W* is the weight (kg), and k1, k2, and k3 are specific parameters used as follows: for males, k1 = 0.3669, k2 = 0.03219, and k3 = 0.1833; for females, k1 = 0.3561, k2 = 0.03308, and k3 = 0.1833. Finally, HBL (mL) was obtained as total blood loss + volume of autologous blood transfusion + volume of ABT − dominant blood loss.

### Statistical analysis

SPSS version 20.0 (IBM Corporation, Armonk, NY, USA) was used for data analysis. For continuous variables with a normal distribution, results were expressed as mean ± standard deviation, and the *t* test was used to compare the differences between the TXA group and the control group. Otherwise, results were expressed as median and 25%–75% interquartile range, and comparisons were performed using the Mann–Whitney U test. Categorical variables were expressed as numbers and percentages, and the Chi-square test was applied for comparisons. The results regarding postoperative Hb and HCT evolution were analyzed by repeated measures analysis of variance. Differences were considered statistically significant for two-sided *P* values < 0.05.

## Results

A total of 996 consecutive patients underwent unilateral primary TKA in single institution between June 2013 and December 2015, of whom 99 patients had preoperative anemia (9.9%). Three patients with moderate or severe anemia were excluded on the basis of our exclusion criteria. A total of 96 patients (14 males and 82 females) were included in our study. The average age was 66.73 years (range, 40–85 years). All baseline characteristics including the age, gender, and preoperative anemia status were comparable (*P *> 0.05) between the control and TXA groups (Table 1).

**Table 1:** Baseline characteristics of preoperatively anemic patients undergoing total knee arthroplasty

	TXA group	Control group	*P*-value
	N = 32	N = 64	
Sex, male/female	7/25	12/52	0.27
Age, years	67.4 ± 10.3	65.5 ± 7.7	0.96
BMI, kg/m^2^	25.5 ± 3.1	26.2 ± 3.8	0.40
ASA score	1.9 ± 0.4	1.8 ± 0.4	0.42
Pre-opCr, μmol/L	65.4 ± 13.9	66.7 ± 9.6	0.58
Pre-opHb, g/dL	11.1 ± 2.6	11.5 ± 4.9	0.17
Pre-opHCT	0.338 ± 0.027	0.341 ± 0.019	0.16
Tourniquet time, min	84.7 ± 26.3	77.7 ± 27.7	0.26
Charlson score	3.00 ± 0.76	3.00 ± 1.49	1.00

Values given as average ± standard deviation, unless otherwise specified

*TXA* tranexamic acid, *BMI* body mass index, *ASA* Society of Anesthesiologists, *Pre-opCr* preoperative levels of creatinine, *Pre-opHb* preoperative levels of hemoglobin, *Pre-opHCT* preoperative levels of hematocrit

**Fig. 1:**
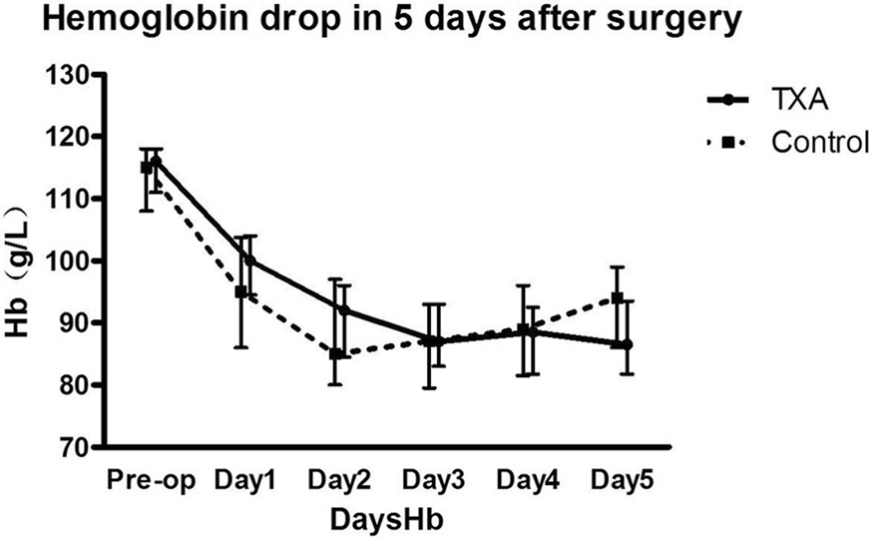
Histogram revealing the hemoglobin (Hb) drop over the first 5 days after total knee arthroplasty. Overall, no statistically significant difference (*P* = 0.763) was noted between the control group and the group of patients who had received tranexamic acid (TXA), although the TXA group showed significantly higher Hb levels on the first postoperative day (9.91 ± 0.6 g/dL vs 9.40 ± 1.1 g/dL; *P *= 0.006)

**Fig. 2:**
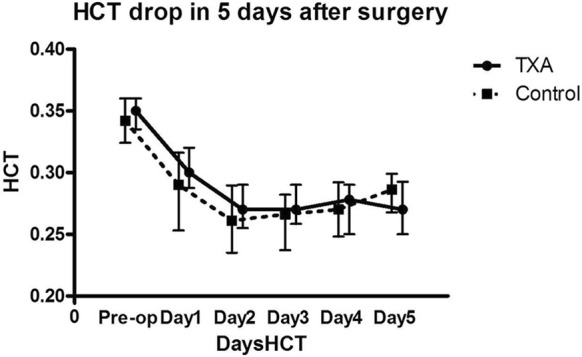
Histogram revealing the hematocrit (HCT) drop over the first 5 days after total knee arthroplasty. No statistically significant difference (*P* = 0.474) was noted between the control group and the group of patients who had received tranexamic acid (TXA)

In the TXA group, the drainage volume and HBL were 200 (100, 320) mL and 182.6 (106.9, 301.7) mL, respectively, compared with 380 (200, 612.5) mL and 283.3 (146.7, 562.3) mL, respectively, in the control group; the difference was statistically significant. The ABT volume was dramatically lower in the TXA group (88.9 ± 230.9 mL) than in the control group (216.3 ± 293.9 mL) (*P *< 0.05; Table 2). Moreover, there were only 4 TXA patients who required ABT, compared to 20 in the control group (*P *< 0.05; Table 3). The Hb levels on the first postoperative day were significantly higher in the TXA group than in the control group (9.91 ± 0.6 g/dL vs 9.40 ± 1.1 g/dL; *P *= 0.006). However, no significant differences between the two groups were noted regarding Hb and HCT levels over the following 4 postoperative days (*P *> 0.05; Figs. 1, 2).

**Table 2:** Effect of TXA administration on blood loss and need for transfusion in preoperatively anemic patients undergoing total knee arthroplasty

	TXA group	Control group	*P-*value
	N = 32	N = 64	
Drainage, mL	200 (100, 320)	380 (200, 612.5)	0.00
Hidden blood loss, mL	182.6 (106.9, 301.7)	283.3 (146.7, 562.3)	0.04
ABT volume, mL	88.9 ± 230.9	216.3 ± 293.9	0.01

Values given as median (25% to 75% interquartile range) or average ± standard deviation

*TXA* tranexamic acid, *ABT* allogeneic blood transfusion

**Table 3:** Chi-square test for the volume of allogeneic blood transfusion and incidence of deep venous thrombosis (DVT) events after total knee arthroplasty in preoperatively anemic patients

	TXA group	Control group	*P*-value
	N = 32	N = 64	
Patients requiring ABT	4 (12.5%)	20 (31.3%)	0.046
Patients with DVT	3 (9.4%)	5 (7.8%)	0.794

Data given as number (percentage)

*TXA* tranexamic acid, *ABT* allogeneic blood transfusion

The incidence of DVT events was low in both groups (3 patients in the TXA group vs 5 patients the in control group), with no statistically significant difference (*P *> 0.05; Table 3).

## Discussion

The overall prevalence of anemia increases with age, and, according to the WHO report [13], amounts to approximately 24% in patients indicated for primary TKA. Therefore, preoperative anemia should be considered as a serious but treatable condition [14]. It was reported that ABT volume after TKA is much higher among preoperatively anemic patients than among normal patients, as are complications and medical costs [15–17]. However, few studies have investigated the effects of TXA in preoperatively anemic patients undergoing TKA. As patients with moderate or severe anemia are typically not indicated for TKA, the treatment for the primary disease should be considered as a priority; for the same reason, such patients were not included in our study.

As an antifibrinolytic agent, TXA has the ability to block fibrinolysin and save fibrinogen, potentially reducing postoperative blood loss and ABT volume after TKA. In a randomized control trial involving 124 patients with normal preoperative hemoglobin levels, Wong et al. [4] found that intraoperative administration of 1.5 g TXA might reduce drainage volume by one-third, and ABT volume to 12.9%. This conclusion was supported by other recent reports.

Our results suggest a similar effect for preoperatively anemic patients, as the drainage volume was lower by almost 50% in the TXA group. The volume of ABT was also significantly decreased by the application of TXA. The condition of preoperatively anemic patients could be considered as an impaired ability to store blood, which contributes to a decreased capability to cope with rapid blood loss after surgery, increasing the need for ABT. The application of TXA reduced the rate of postoperative blood loss, as suggested by the histogram in Fig. 1, which revealed that the hemoglobin levels dropped faster in the control group over the first 2 days after surgery, although the difference between the groups was statistically significant only for the first postoperative day. Therefore, administration of TXA helped the patients tolerate the postoperative hemoglobin drop, which explains the encouraging results noted in our study.

The histogram suggested higher HCT levels in the TXA group, repeated measures analysis indicated there was no significant difference between the two groups, and the Hb and Hct levels were higher at day 5 in the control group. The authors believe that the higher ABT volume in the control group should be considered as an influence factor in the analysis of the Hb and HCT levels. Therefore, further study should perform a subgroup analysis for the patients who received ABT. Additionally, it should be noted that the intravenous TXA dose of 1 g could typically be eliminated up to 90% via the kidneys within 24 h, with a half-life of 2–3 h in vivo. This might explain why only the drainage and Hb on the first postoperative day had been significantly decreased in TXA group, since the drug effect could not last during the following days.

HBL typically continues for several days after surgery, and might contribute 50% of the total blood loss [18]. In a randomized control trial study reported by Good et al. [19], HBL was reduced by TXA to 524 (330 ± 962) mL. The results obtained in the current study suggest that the improvement caused by TXA could be very significant. Considering of the PK of TXA [20], the HBL might be additionally decreased if more TXA could be administered under a safer protocol with prolonged application. Aiming to achieve this goal, a randomized control trial study is being undertaken by our research team.

Although several DVT events associated with TXA usage have been reported previously, plenty of studies have confirmed the safety of TXA application under the protocol described in the present study. In a meta-analysis of 15 randomized clinical trials, Zhang et al. [21] reported that TXA did not increase the risk of DVT, which is in agreement with many other reports. Indeed, we found that the incidence of DVT events was comparable between the TXA and control group, which suggests that TXA can be used safely to control blood loss in preoperatively anemic patients.

Considering the potential higher risk of thrombosis, nausea and vomiting, we only administrated TXA 1.0 g 15 min before inflation of the tourniquet, and 1.0 g after tourniquet release postoperatively in this study. The safety and risk of the daily and local administration of TXA need further investigation.

## Conclusion

We concluded that TXA have a positive role for patients with preoperative anemia in primary total knee arthroplasty. The significance of this study is that it analyzed, for the first time, the efficiency of TXA in TKA patients with mild preoperative anemia, and noted that the application of TXA could decrease the ABT and HBD volume without increasing the DVT risk. In addition, our preliminary results suggest that TXA appears efficient mainly on the first postoperative day. Nonetheless, as the intrinsic feature of a retrospective study, the present investigation may be considered only as a preliminary assessment, and further study is warranted.

## Data Availability

All data generated or analyzed during this study are included in this published article.
